# A Lightweight Dual-Stream Network with an Adaptive Strategy for Efficient Micro-Expression Recognition

**DOI:** 10.3390/s25092866

**Published:** 2025-05-01

**Authors:** Xinyu Liu, Ju Zhou, Feng Chen, Shigang Li, Hanpu Wang, Yingjuan Jia, Yuhao Shan

**Affiliations:** 1 College of Electronic and Information Engineering, Southwest University, Chongqing 400715, China; liu1223xy@email.swu.edu.cn (X.L.); zhouju@email.swu.edu.cn (J.Z.); chenf031448@email.swu.edu.cn (F.C.); whp012022333000496@email.swu.edu.cn (H.W.); jyj151005@email.swu.edu.cn (Y.J.); 2 Chongqing Key Laboratory of Generic Technology and System of Service Robots, Southwest University, Chongqing 400715, China; 3 Institute of Legal Psychology and Intelligent Computing, Southwest University, Chongqing 400715, China; 4 Shenzhen Institute of Advanced Technology, Chinese Academy of Sciences, Shenzhen 518055, China; 5 School of Information Science, Hiroshima City University, Hiroshima 7313194, Japan; shigangli@hiroshima-cu.ac.jp

**Keywords:** micro-expression recognition, motion magnification, optical flow, adaptive strategy, lightweight model, deep learning

## Abstract

Micro-expressions (MEs), characterized by their brief duration and subtle facial muscle movements, pose significant challenges for accurate recognition. These ultra-fast signals, typically captured by high-speed vision sensors, require specialized computational methods to extract spatio-temporal features effectively. In this study, we propose a lightweight dual-stream network with an adaptive strategy for efficient ME recognition. Firstly, a motion magnification network based on transfer learning is employed to magnify the motion states of facial muscles in MEs. This process can generate additional samples, thereby expanding the training set. To effectively capture the dynamic changes of facial muscles, dense optical flow is extracted from the onset frame and the magnified apex frame, thereby obtaining magnified dense optical flow (MDOF). Subsequently, we design a dual-stream spatio-temporal network (DSTNet), using the magnified apex frame and MDOF as inputs for the spatial and temporal streams, respectively. An adaptive strategy that dynamically adjusts the magnification factor based on the top-1 confidence is introduced to enhance the robustness of DSTNet. Experimental results show that our proposed method outperforms existing methods in terms of F1-score on the SMIC, CASME II, SAMM, and composite dataset, as well as in cross-dataset tasks. Adaptive DSTNet significantly enhances the handling of sample imbalance while demonstrating robustness and featuring a lightweight design, indicating strong potential for future edge sensor deployment.

## 1. Introduction

Different from macro-expression, micro-expressions (MEs) are fleeting, subtle facial movements that occur when people unconsciously reveal their genuine emotions while attempting to suppress them [1,2]. Accurately capturing and recognizing MEs can provide insight into a person’s genuine emotions. Therefore, ME recognition is of great significance in social interactions [3], medical treatment [4], lie detection [5], and national security [6].

As a complex emotional expression, MEs are difficult to elicit due to their short duration and weak facial muscle movements. Furthermore, the acquisition of ME data relies on high-frame-rate cameras as primary sensors to capture these transient phenomena, resulting in substantial data collection difficulties and consequent scarcity of relevant data resources. These characteristics of MEs pose a challenge for accurate ME recognition. Currently, to promote studies on ME recognition, researchers commonly follow a three-step process [7]: preprocessing, MEs generation, and recognition model.

In the preprocessing, various techniques are employed to eliminate irrelevant factors from the original ME video, thereby enhancing the recognition performance. Among them, face detection and face alignment are the most common preprocessing methods [8,9]. Face detection aims to remove the background and keep only the face; face alignment reduces the effects of changes in facial shape and pose through aligning facial keypoints and using affine transformations. Additionally, motion magnification, as a unique preprocessing technique, addresses the problem of the low intensity of ME muscle movements by magnifying them [10,11], which contributes to improving recognition accuracy. However, most motion magnification methods are linear, exhibiting poor robustness and susceptibility to noise. They struggle to effectively magnify subtle and complex muscle movements.

In the MEs generation, the Generative Adversarial Network (GAN) [12] has been a prevailing method widely used by lots of researchers. The ULME-GAN [13] generates controllable ME sequences by re-encoding the action unit (AU) matrix, and it has been proven on public datasets that the generated training data can enhance the performance of recognition models. In  the literature [14], two independent GAN models are employed to generate horizontal and vertical optical flow images, thereby expanding the training data for classification models. Additionally, the First Order Motion Model (FOMM) [15] and temporal interpolation technique [16] have also been applied to the MEs generation and data augmentation. However, due to the lack of MEs datasets and insufficient data for training ME generation networks, the quality of generated ME images still falls short of the desired level.

In the recognition model, the characteristics of low intensity and short duration make the ME recognition task highly challenging. Depending on the input, it is typically divided into three technical approaches: image, video, and optical flow. Among these, the image-based method often employs apex frame analysis [17], which simplifies processing but sacrifices temporal information. Video and optical flow inputs, capable of capturing temporal dynamics, are also crucial in ME recognition. Optical flow methods encompass sparse optical flow and dense optical flow. Sparse optical flow typically calculates the primary direction of localized muscle movements for MEs [18], but may overlook subtle motions. Dense optical flow calculates the global optical flow variation from the onset frame to the apex frame [19], even yielding a histogram of optical flow directions for different regions on the face, thereby extracting temporal information of MEs. However, dense optical flow comes with excessive computational load. In contrast to optical flow, when video is used as an input, it is an end-to-end approach that extracts both spatial and temporal information and can be directly processed without additional operations [20]. Nonetheless, when the input is video, it still faces the challenge of large computational costs. Furthermore, current ME recognition models exhibit limited robustness when applied to cross-dataset recognition tasks.

In summary, there are the following issues with ME recognition: (1) The mainstream method for motion magnification is mainly linear magnification, which exhibits poor robustness and is susceptible to noise interference; (2) ME generation networks struggle to generate controllable MEs, and ME data are still insufficient; (3) Sparse optical flow with the ability to capture temporal information tends to ignore subtle muscle movements, while dense optical flow and video have high computational complexity; (4) The existing recognition models are deficient in robustness.

To solve the above issues and fully leverage network characteristics, this study carefully designs feature extraction methods and network architectures aimed at developing a lightweight model for efficient ME recognition. Firstly, to solve issue (1), we employ a motion magnification network based on transfer learning. This method not only improves the robustness compared to the traditional linear magnification but also effectively addresses the problem of insufficient training data for the magnification network. To address issue (2), a magnification factor is introduced to control the degree of motion magnification. By adjusting this factor to obtain ME images with different magnification levels, we successfully expand the data for training the recognition model. For issue (3), we employ the recurrent all-parallel field transform (RAFT) [21] to calculate the correlation of each pixel point between the onset frame and the magnified apex frame to obtain the magnified dense optical flow (MDOF). This approach significantly reduces the computational effort while still capturing the feeble muscle motion of MEs. Subsequently, we design a dual-stream spatio-temporal network (DSTNet) to extract spatial features from the magnified apex frame and temporal features from the MDOF. For issue (4), we incorporate an adaptive mechanism into DSTNet, which dynamically adjusts the magnification factor based on the top-1 confidence output via the model, thus, enhancing its robustness. Our proposed method demonstrates superior performance on three public datasets and composite datasets, especially when dealing with imbalanced datasets where its F1-score is particularly outstanding. In addition, the method also exhibits excellent performance in cross-dataset recognition tasks, demonstrating the robustness of the recognition model. While effectively addressing various issues faced by ME recognition, the adaptive DSTNet also possesses lightweight characteristics, significantly enhancing recognition efficiency in practical applications. The main contributions of this paper are as follows:

- The utilization of transfer learning-based motion magnification network effectively amplifies feeble motion of facial muscles in MEs and also expands the training dataset.
- The pixel-level dense optical flow MDOF is innovatively designed to extract only once from the magnified apex frame. This design not only maintains the accurate capture of subtle facial muscle movements but also significantly reduces computational complexity.
- DSTNet with an adaptive strategy is constructed to extract deep representations from spatio-temporal information for ME recognition. Experimental results on various tasks demonstrate its effectiveness.

The paper is organized as follows: Section 2 introduces the related work on ME recognition. Section 3 details the proposed adaptive DSTNet and its module. Section 4 presents the ablation experiments, recognition results on three tasks, and comparisons with other methods. Section 5 summarizes and discusses this study.

## 2. Related Work

MEs often reveal the genuine emotions of human beings, so the task of researching ME recognition is becoming more and more important. However, due to the subtle and fleeting characteristics of MEs, simple processing methods often fail to achieve better performance. With the development of computer hardware and deep learning, the use of neural networks has greatly improved the accuracy of ME recognition. One of the most common approaches in ME recognition is the single-feature input strategy. This involves designing a high-performance deep network architecture tailored to the characteristics of different input methods. Given the complementarity between different information, another commonly-used approach is to employ a multi-feature input strategy [22,23], where various sub-streams are utilized to extract deep features from image, video, and optical flow inputs, respectively. These features are then fused and processed to enhance accuracy. For example, there are methods that learn facial features and AU matrices for ME recognition, where facial features refer to connecting facial feature points to form a graph containing node values and edge weights [24]. Despite their success, these methods often prioritize accuracy over computational efficiency, leading to complex models with high resource demands. This motivates our investigation of efficient feature representation that maintains discriminative power while reducing resource consumption.

Another critical challenge in ME recognition stems from the limited and imbalanced training data, which significantly compromises deep network performance. Although GANs have been employed to synthesize additional training samples, their effectiveness for MEs remains fundamentally constrained by both the subtle nature of facial muscle movements and the inherently small dataset scale [25]. Furthermore, FOMM-based techniques demonstrate insufficient AU controllability while being further limited by the scarcity of source ME videos for driving large-scale generation [26]. These compounded challenges highlight the urgent need for more efficient data augmentation solutions to prompt ME recognition with enhanced accuracy and real-world applicability.

Transfer learning is a method of transferring knowledge from a source task to a target task to improve learning of the target task. It has been widely used to solve the problem of applying complex networks to small datasets [27]. Currently, motion magnification networks are primarily trained on macro-expression datasets, whereas ME datasets are relatively smaller. Therefore, in this study, we introduce a transfer learning approach to the motion magnification network. We use large-scale datasets of macro-expressions to initialize the weights, and then fine-tune these weights on smaller ME datasets to ensure that the motion magnification network can effectively magnify subtle facial muscle movements in MEs. Importantly, this magnification process simultaneously serves as a data augmentation mechanism, generating enhanced training samples while improving feature extraction, thereby addressing two critical challenges in ME recognition simultaneously.

While the field of ME recognition has seen significant progress, with various sophisticated methods being proposed to improve performance, one critical aspect that has often been overlooked is the model’s complexity and computational requirements. Many existing approaches focus solely on maximizing recognition accuracy, employing deep neural networks with hundreds of millions of parameters and requiring substantial computational resources. This trend towards more complex models not only increases the computational burden but also limits their practical applications. In this study, we combine network structure design to address the various challenges faced by MEs with reduced computational resources. By meticulously designing a lightweight model and incorporating the adaptive strategy, we achieve a reasonable balance between recognition performance and model scale.

## 3. Proposed Method

### 3.1. Overview of Adaptive DSTNet

**Figure 1:**
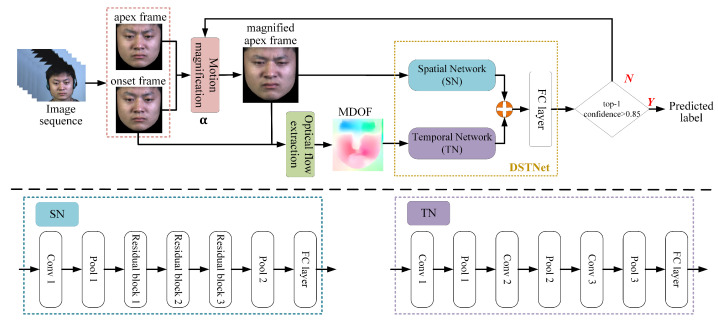
The framework of adaptive DSTNet for ME recognition.

In this study, we propose a lightweight dual-stream spatio-temporal network with an adaptive strategy, named adaptive DSTNet, for efficient ME recognition. This approach specifically addresses the challenges posed by the subtle facial movements, short duration, and limited data samples inherent in MEs. The algorithmic framework of this method is illustrated in Figure 1.

The adaptive DSTNet framework processes onset and apex frames extracted from publicly available ME datasets using their provided temporal annotations. It amplifies the motion of the apex frame to enhance facial muscle movements, which are then fed into a Spatial Network (SN) to extract spatial features representing facial muscle state. Additionally, these amplified frames serve as augmented samples for the training dataset, mitigating the issue of limited ME data. MDOF is extracted between the onset and magnified apex frames for further processing via a Temporal Network (TN) to capture temporal dynamics of facial movement trends. DSTNet deeply extracts and fuses spatio-temporal features to achieve ME recognition. To enhance model robustness, an adaptive strategy is implemented: if the top-1 confidence level is below 0.85, the magnification factor α is increased, and the images are reprocessed to extract new magnified apex and MDOF for re-recognition. This iterative process continues until the top-1 confidence exceeds 0.85 or a predefined termination condition is met. This approach effectively represents muscle movement states and trends in ME sequences, and the integration of the dual-stream network with the adaptive strategy significantly improves ME-related feature representation, thereby enhancing recognition performance.

### 3.2. Motion Magnification

**Figure 2:**
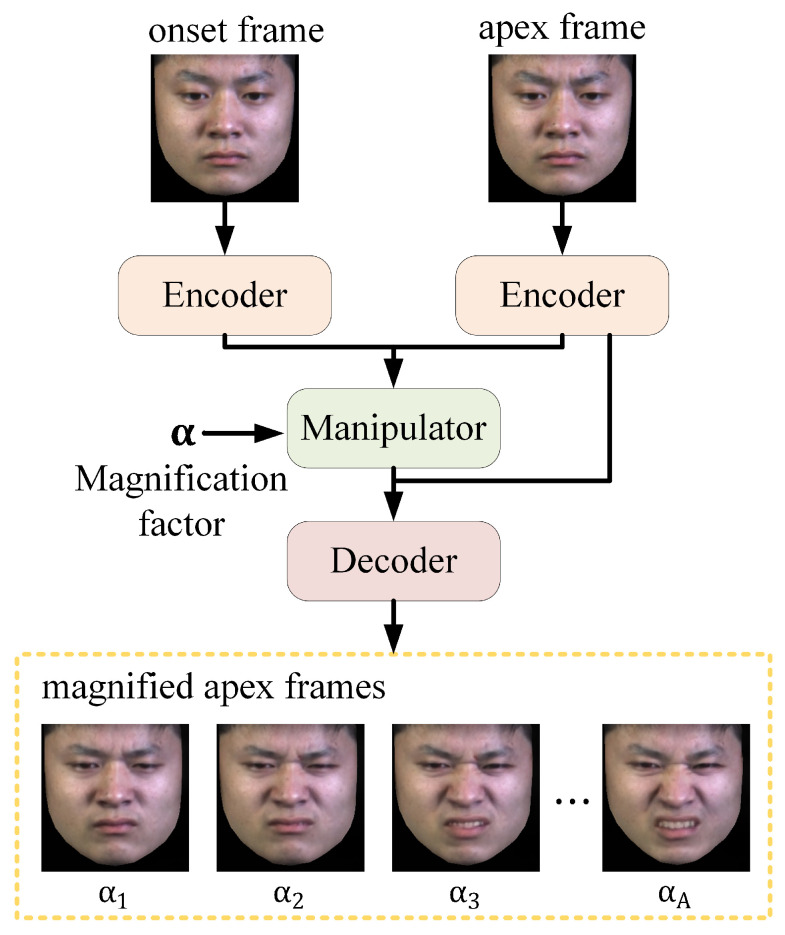
Motion magnification structure. The magnified apex frames with different factors are shown in the yellow dashed box, with the subscript indicating the value of α.

Because the motion of ME is very feeble, it is difficult for it to be recognized by computers. To solve this issue, the study employs a deep learning-based motion magnification network to magnify ME facial muscle movements. As shown in Figure 2, the network consists of three main parts: encoder, manipulator, and decoder.

The encoder serves as a spatial decomposition filter, extracting shape features $S=G_{\mathrm{shape}\;}\left(x\right)$ and texture features $T=G_{\mathrm{texture}\;}\left(x\right)$ from a single frame. In the manipulator, a magnification factor α is introduced. By calculating the difference in shape features between two given frames (the onset frame *a* and the apex frame *b*) and multiplying it by α, the magnified shape feature $G_{s}\left(S_{a},S_{b},\alpha \right)$ can be obtained. The calculation is as follows:

(1) $$G_{s}\left(S_{a},S_{b},\alpha \right)=S_{a}+h\left(\alpha \cdot g\left(S_{b}-S_{a}\right)\right)$$

where g(·) represents a 3×3 convolution and ReLU, and h(·) represents a 3×3 convolution and a 3×3 residual block.

Different from other motion magnification models, this study generates a single magnified apex frame, independent of temporal factors. Consequently, the decoder simplifies the time-related processing component. It primarily fuses inputs from the encoder and manipulator, superimposing the magnified shape features onto the onset frame texture features $T_{a}$ to generate the magnified apex frame $\tilde{b}$. This means that the output magnified apex frame can be controlled solely by adjusting the magnification factor. The calculation is as follows:

(2) $$\tilde{b}=\tilde{I}\left(\alpha \right)=f\left(T_{a}+G_{s}\left(S_{a},S_{b},\alpha \right)\right)$$

where f(·) represents the function that reconstructs the texture features with the magnified shape features, and $\tilde{b}$ represents the magnified apex frame.

Transfer learning enables deep CNNs to be applied to small ME datasets by pre-training on larger macro-expression datasets. In this study, we selected 10,431 images from four macro-expression datasets: CK+ [28], Oulu-CASIA NIR&VIS [29], Jaffe [30], and MUGFE [31], expanding each category to 5000 images to form a new dataset for transfer learning. The magnification network is pre-trained on this expanded dataset and then fine-tuned on ME datasets: SMIC [32], CASME II [33], and SAMM [34]. As shown in the yellow dashed box in Figure 2, using a disgust sample from CASME II [33], a notable change in expression amplitude is observed as the magnification factor α increases from 1 to *A*. This change is particularly evident in the disgust-related action units: AU4 (brow lower), AU9 (nose more wrinkled), and AU10 (upper lip raised).

Magnifying the facial muscle movements has a dual benefit: it emphasizes the distinct patterns of MEs, and generates more apex frames by adjusting different magnification factors, effectively expanding data for training. The developed motion magnification network not only enhances subtle muscle movements but also increases the perceptibility of MEs that would otherwise be lost due to sensor limitations (e.g., motion blur under low illumination).

### 3.3. MDOF

Magnifying facial muscle movements alone has limitations for recognition due to varying initial states. Therefore, extracting the overall trend of movements from ME sequences is necessary. Traditional optical flow methods struggle with subtle ME movements. Thus, we introduced RAFT [21] to capture these movements accurately. For real-time extraction, RAFT is applied to the onset and magnified apex frames, yielding MDOF to characterize movement trends.

In this study, the MDOF is obtained by first extracting the feature vectors for each pixel in the onset frame *a* and the magnified apex frame $\tilde{b}$. These feature vectors are denoted as $g_{\theta }\left(a\right)\in R^{H\times W\times D}$ and $g_{\theta }\left(\tilde{b}\right)\in R^{H\times W\times D}$, where *H*, *W*, and *D* represent the height, width, and depth of the feature maps, respectively. To reduce the computational load, the feature extraction network $g_{\theta }\left(\cdot \right)$ is designed as a multi-layer network with shared weights. Subsequently, the dot product is used to calculate the correlation between any two points, thereby obtaining a correlation matrix that contains information about all the pixels:

(3) $$C_{ijkl}=\sum_{h}g_{\theta }{\left(a\right)}_{ijh}\cdot g_{\theta }{\left(\tilde{b}\right)}_{klh}$$

where $C_{ijkl}\in R^{H\times W\times H\times W}$ represents the correlation matrix, *i*, *j* and *k*, and *l* are spatial index positions in the feature maps, and *h* represents channel index of the feature maps.

Next, a multi-scale 4D correlation pyramid $\left\{C^{1},C^{2},C^{3},C^{4}\right\}$ is constructed using pooling operations. This pyramid captures multi-scale image similarity features, providing key information for pixel displacement. Finally, a recurrent mechanism based on the Gated Recurrent Unit (GRU) is employed to update the optical flow [21]. Specifically, in conjunction with the current optical flow estimate, relevant features are retrieved from the correlation pyramid to update the optical flow. This process can be simplified and represented as:

(4) $$f^{\left(t+1\right)}=\mathrm{GRU}\left(f^{\left(t\right)},\mathrm{lookup}\left(C,f^{\left(t\right)}\right)\right)$$

where $f^{\left(t\right)}$ denotes the optical flow estimate at the *t*-th iteration, $\mathrm{lookup}(C,f^{\left(t\right)})$ represents the correlation values retrieved from the correlation pyramid based on the current optical flow estimate, and $f^{\left(t+1\right)}$ denotes the updated optical flow. Through this pixel-by-pixel correlation lookup and optical flow update process, we achieve high-precision optical flow estimation, thereby obtaining the MDOF that accurately reflects subtle muscle motion changes of MEs.

**Figure 3:**
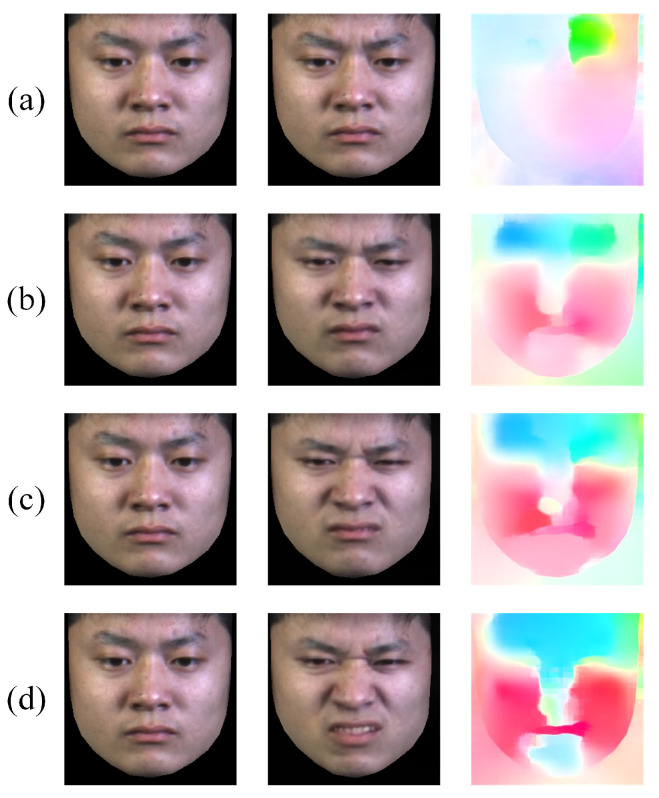
Visualization of the onset frames (**left**), the apex frames (**middle**), and corresponding optical flow images (**right**) for a disgust sample in CASME II [33]. (**a**) is the original apex frame and dense optical flow (α=0), and (**b**–**d**) are magnified apex frames and MDOFs produced via the sequentially enhanced α (α=1,2,3).

As shown in Figure 3, the first column presents onset frames, the second column presents original apex frame (α=0) and magnified apex frames with varying α (α=1,2,3), and the third column visualizes original optical flow (computed between onset and original apex frame) and the MDOFs (computed between onset and magnified apex frames). Darker colors in optical flow indicate more intense motion. By observing the original dense optical flow extracted from the onset frame and the original apex frame, it can be noted that although it successfully captures the motion feature of AU4, the capture of motions in other regions is less satisfactory. However, when the MDOF is extracted for the magnified apex frames (the last three rows of the third column in Figure 3), a clear similarity in motion patterns emerges, indicating that all facial muscle movements associated with the expression of disgust are effectively captured, such as AU9 (nose wrinkler) and AU10 (upper lip raiser). Furthermore, as the magnification factor increases (from upper to lower), the visualized images of MDOF become darker, providing richer information for the model to more accurately recognize MEs. This feature extraction method effectively standardizes raw sensor outputs into unified spatio-temporal representations, thereby significantly facilitating subsequent ME analysis.

### 3.4. DSTNet

DSTNet consists of SN and TN, which represents a spatial stream network and a temporal stream network, respectively. The details of DSTNet are shown in Table 1. SN extracts multi-level features from the magnified apex frame using convolutions, employing residual blocks to mitigate gradient vanishing. TN uses a shallow convolutional layer to capture subtle temporal features from MDOF input, reducing computational load. The average pooling is to reduce the dimension of feature output, effectively mitigating the influence of irrelevant data on model training. The FC layer integrates the features of the previous layer and improves the model’s nonlinear fitting performance. Subsequently, the features output via SN and TN are fused, and the top-1 confidence level is output for subsequent adaptive adjustment and ME recognition.

In the field of ME recognition, the trade-off between computational efficiency and recognition performance is particularly crucial. By concentrating the computational load on motion magnification and MDOF calculation, DSTNet features as high computational efficiency and performance. It requires only 64.77 M floating point operations (FLOPs) and 3.15 M parameters to achieve outstanding recognition capabilities for three-channel 256×256 ME images. This lightweight architecture offers the feasibility of implementing real-time inference on edge computing devices, allowing it to constitute a complete real-time ME analysis system together with high frame rate imaging sensors. The subtle facial muscle movements of MEs pose a significant challenge for deep networks in capturing key features. However, our proposed method is capable of extracting and utilizing these information more directly and rapidly.

### 3.5. Adaptive Strategy

To enhance ME recognition performance, we introduced an adaptive magnification factor adjustment algorithm into the DSTNet framework. This strategy initiates after obtaining the top-1 confidence score by using the DSTNet to recognize the magnified apex frame and its corresponding MDOF. If the current confidence exceeds the previous highest, the model updates its highest confidence and recognition result. If it meets or exceeds the preset threshold (set at 0.85 in this study), the loop terminates without further magnification, and results are output. During iteration, the magnification coefficient α increases until reaching the maximum (set at seven in this study). Ultimately, the algorithm outputs the highest confidence and corresponding result. The detailed algorithm flow is presented in Algorithm 1.
**Algorithm 1:** Adaptive adjustment strategy for DSTNet**Input**: ME image sequence: img_seqinitial magnification factor: init_αconfidence threshold: conf_threshmaximum magnification factor: max_α**Output**: top-1 confidence: high_confRecognition result: result**Initialize: **curr_α←init_αhigh_conf←0.0result←Null
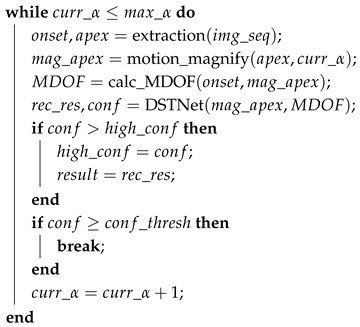
**return **high_conf,result

Through this adaptive adjustment mechanism, the motion magnification factor can be dynamically adjusted according to the difficulty of ME recognition task, thereby improving the accuracy of the recognition model. When dealing with challenging recognition tasks, such as cross-dataset ME recognition, it can adaptively enhance subtle motion features and fully utilize spatial and temporal information for precise recognition, thereby enhancing the model’s robustness and enabling it to better handle various complex scenarios. It is worth noting that this adaptive strategy does not increase the model’s parameters or FLOPs.

## 4. Experimental Results and Analysis

### 4.1. Datasets and Parameter Settings

Experiments were conducted on SMIC [32], CASME II [33], and SAMM [34] with 164, 246, and 136 samples respectively. Among them, SMIC is a three-class problem, while CASME II and SAMM are both five-class problem. In addition, we also conducted experiments on the dataset of the Composite Dataset Evaluation (CDE) of the Micro-Expression Grand Challenge 2019 (MEGC 2019) [35], which incorporates SMIC, CASME II, and SAMM data, totaling 442 samples in three classes.

During model training, unified parameters were used: DSTNet with a learning rate = 0.001, weight decay = 0.0005, batch size = 4, epochs = 50, Adam optimizer, and cross-entropy loss. Magnified apex frames were generated by adjusting different α values to ensure that each category contains 150 samples. In the adaptive α adjustment, confidence threshold = 0.85, initial α = 3, and max α = 7. Notably, DSTNet parameters remain unchanged when applying the adaptive strategy. The adaptive strategy is only used in the composite dataset and cross-dataset recognition.

### 4.2. Validation Methods and Evaluation Metrics

In this study, we addressed three types of classification problems: single dataset, composite dataset, and cross-dataset recognition. We followed ME field conventions and primarily used two cross-validation methods to evaluate recognition models [35].

The first method was Leave-One-Subject-Out (LOSO), which divides the dataset by the subjects. In each validation, one subject’s data is used as the test set, and the rest as the training set. This process cycles until all data is tested. LOSO is applicable to single and composite dataset recognition. The second method was Leave-One-Dataset-Out (LODO), where one dataset is reserved as the test set, and all others as the training set. This process repeats until each dataset is tested. LODO evaluates the model’s generalization across datasets and is used for cross-dataset recognition.

For single dataset tasks, we assessed performance using accuracy (Acc) and F1-score (F1-score). For the composite dataset tasks, MEGC 2019 requires unweighted F1-score (UF1) and unweighted average recall (UAR) [35]. For cross-dataset tasks, we adopted Acc, UAR, and UF1 for comparison with other methods.

### 4.3. Ablation Analysis

This section mainly conducts ablation experiments on the proposed motion magnification method, MDOF extraction method, and the DSTNet structure. All experiments were performed on the five-class classification task of CASME II.

#### 4.3.1. Motion Magnification Visualization of Transfer Learning Ablation

**Figure 4:**
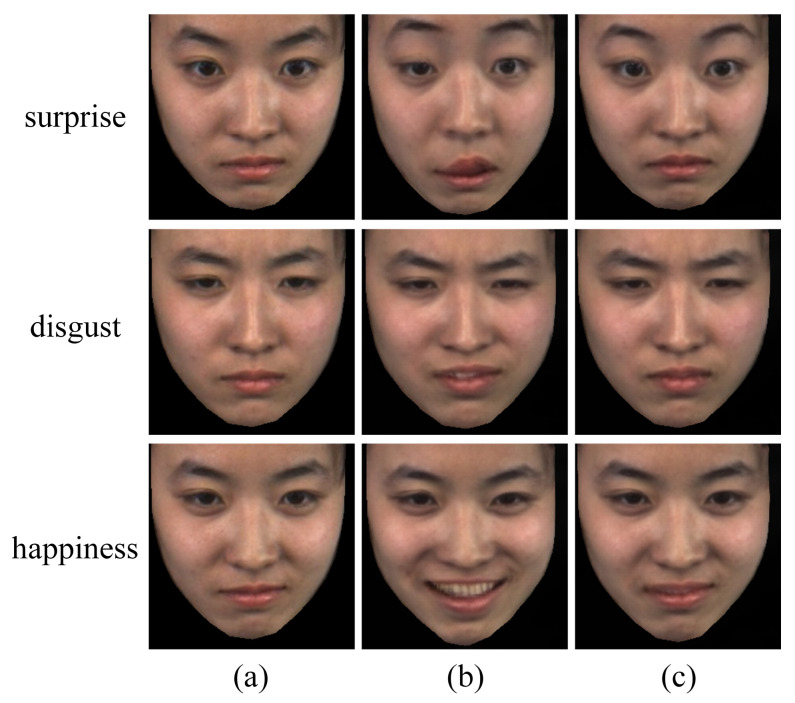
Visualization comparison of motion magnification effects: (**a**) original apex frames; (**b**) magnified apex frames without transfer learning; (**c**) magnified apex frames with transfer learning.

To validate the effectiveness of our proposed transfer learning-based motion magnification method, we performed an ablation analysis on the motion magnification network regarding the use of transfer learning. As shown in Figure 4, while both motion magnification approaches successfully enhance facial muscle movements, distinct differences emerge in their magnification quality. The non-transfer-learning-based magnification (second column) results in unnatural over-magnification of facial muscle movements, distorting the subtle characteristics of MEs and even introducing artifacts (e.g., the mouth region in the surprise expression). In contrast, our transfer learning strategy—pre-trained on macro-expressions and fine-tuned on MEs—enables the motion magnification network to achieve more precise magnification (third column). This approach neither exaggerates nor misses subtle facial dynamics, effectively preserving the subtle facial patterns of MEs.

#### 4.3.2. Optical Flow Visualization of Different Methods

**Figure 5:**
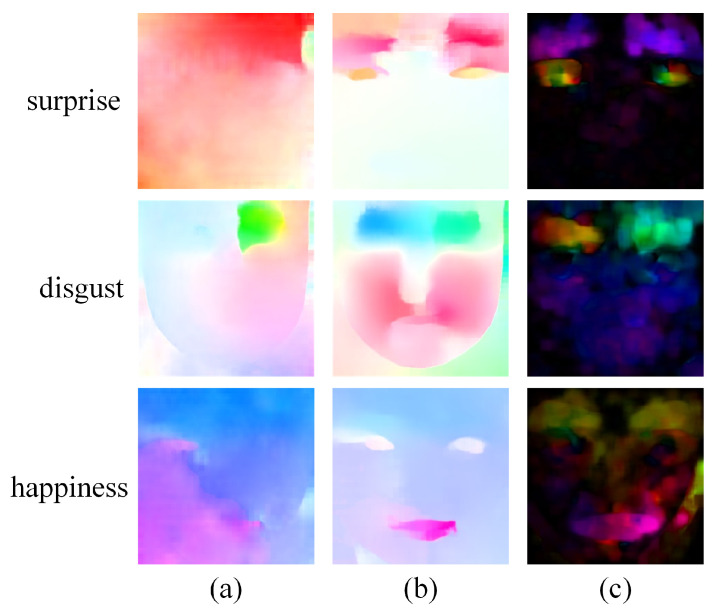
Visualization comparison of optical flow fields extracted using different methods: (**a**) RAFT between original apex and onset frames; (**b**) RAFT between magnified apex and onset frames, i.e., MDOF; (**c**) Sparse optical flow between magnified apex and onset frames.

This study designed MDOF to characterize temporal dynamics in MEs, which is extracted via the dense optical flow method RAFT between motion-magnified apex frames and onset frames, effectively enhancing subtle motion information in MEs. To validate the effectiveness of MDOF, we compare other optical flow extraction methods. As shown in Figure 5, the original RAFT optical flow (first column), derived from the original apex and onset frames, can feature facial region motion but fails to distinguish between ME categories clearly. In contrast, the proposed MDOF (second column) effectively captures crucial muscle movements in different ME categories, such as AU5 (upper lid raiser) in surprise, AU4 (brow lowerer) and AU9 (nose wrinkler) in disgust, as well as AU12 (lip corner puller) in happiness. The sparse optical flow method (third column) demonstrates poor suppression of non-motion regions, introducing considerable noise. These results indicate that MDOF doubly optimizes ME representation by employing a more refined dense optical flow extraction method on magnified apex frames. Without substantially increasing computational cost, MDOF not only highlights motion pattern differences between categories but also suppresses irrelevant noise, thereby providing more discriminative temporal features for improving model performance.

#### 4.3.3. α Ablation

**Figure 6:**
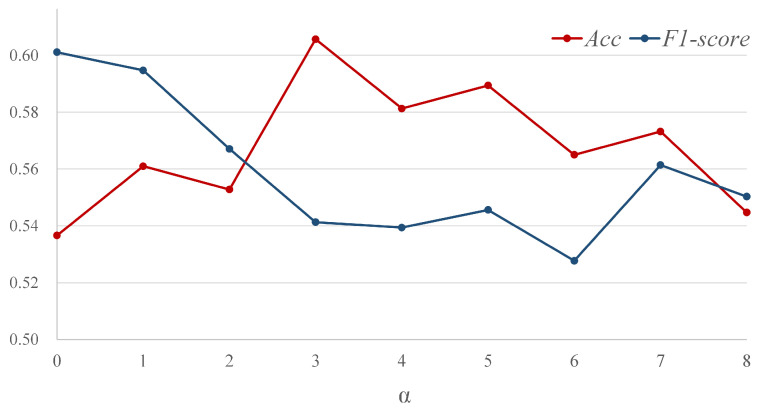
Results of the magnified apex frames with different α on SN, where α=0 indicates using original apex frame.

To explore the optimal range of the factor α in the motion magnification network, we feed magnified apex frames with varying degrees into the single-stream network SN for validation. As shown in Figure 6, when α is set to 0, indicating no motion amplification and the use of original apex frames as input, the model’s Acc is relatively low. As α increases, there is a notable upward trend in Acc, peaking at α=3. However, when α exceeds 7, both the Acc and F1-score of the model decline. Therefore, we chose to expand the training dataset within the α range of 3 to 7 in subsequent experiments.

#### 4.3.4. Input Ablation

We conducted an input ablation analysis to verify the enhancing effect of the proposed motion magnification network and MDOF on ME recognition, which served as inputs for SN and TN, respectively. In this experiment, the magnification factor α was set to 3, and the experimental results are presented in Table 2.

In the first set of experiments, compared to the original apex frames, the magnified apex frames increase the model’s Acc by approximately 7%. In the second set of experiments, using the MDOF extracted from magnified apex frames raises the Acc to 0.6179, and the F1-score also experiences a significant improvement. The experimental results fully demonstrate that the motion magnification network based on transfer learning can effectively enhance the information of facial muscle movement states, and the designed MDOF can capture the motion trends of facial muscles evolution. These enhancements make the pattern differences of MEs more pronounced, thereby improving the recognition performance.

#### 4.3.5. Data Balance Ablation

When employing the motion magnification network to magnify apex frames, this process can also increase the samples to balance the training dataset. As demonstrated in Section 4.3.3, in this ablation experiment, for the imbalanced dataset, we only adopted α=3 to magnify ME samples, with the sample size remaining consistent with the original dataset. For a balanced dataset, we generated magnified apex frames by setting different α values (α=3,4,5,6,7) to ensure that each category contains 150 samples. The experimental results are presented in Table 3.

Compared to training with an imbalanced dataset, using a balanced dataset to train the model reduces the gap between Acc and F1-score. In terms of F1-score, the “magnified apex + SN” combination after data balancing achieves a 6.34% improvement over the imbalanced dataset; this improvement is even more pronounced using the “MDOF + TN” method, reaching 9.19%. The above findings suggest that the model’s recognition performance across various categories has been well improved, thereby confirming that the issue of data imbalance is effectively alleviated. Additionally, this also enables the model to learn to capture the facial muscle movements of MEs under different magnification factors, laying the foundation for subsequent adaptive magnification strategy.

#### 4.3.6. Backbone Depth Ablation

To preserve the lightweight characteristics of DSTNet, this study employed an efficient shallow ResNet architecture for the SN and a basic CNN structure for TN. While keeping the backbone architecture fixed, we systematically investigated the impact of network depth on recognition performance. The ablation analysis validates the recognition performance of using two to four residual blocks in the SN and three to five convolutional layers in the TN. The experimental results are presented in Table 4.

The experimental results demonstrate that the model achieves optimal recognition Acc and F1-score when SN contains three residual blocks and TN adopts three convolutional layers. Under this configuration, DSTNet maintains its lightweight advantage, requiring only 64.77 M FLOPs and 3.15 M parameters. Given our preprocessed input data (magnified apex and MDOF) specifically designed for micro-expression recognition, these shallow network architectures prove sufficient for extracting key discriminative features. The performance analysis confirms that deeper networks do not guarantee improved results, as they may induce overfitting while increasing computational demands.

#### 4.3.7. Network Structure Ablation

In DSTNet, both the spatial and temporal networks share the same depth, with the difference lying in: the SN features residual connections to process spatial information across different scales, while the TN employs a traditional convolutional structure without residual blocks. Therefore, we designed relevant ablation experiments to demonstrate the effectiveness of the structural setup of DSTNet. All experiments used a balanced training dataset, as demonstrated in Section 4.3.5, and the experimental results are shown in Table 5.

The experimental results indicate that the dual-stream network structure generally exhibits advantages over the single-stream network in ME recognition. Specifically, the results of the third row (“magnified apex + SN, magnified apex + TN”) show that indiscriminately inputting the same input into different types of networks may actually impair model performance. Similarly, the results of the fourth row also indicate that, although a dual-stream network structure is adopted, using MDOF as input for both networks results in an Acc of 0.6911, which is only slightly higher than that of “MDOF + single-stream TN”. These results suggest that merely adopting a dual-stream structure is not enough to guarantee performance improvement; the selection of input data for different networks is also crucial. In the last row of experiments, namely the DSTNet proposed in this paper, the Acc reaches its highest at 0.7398, and the F1-score also reaches its highest at 0.7424. This validates the effectiveness of the DSTNet designed for specific inputs in ME recognition.

### 4.4. Results Comparison with Other Methods

#### 4.4.1. Single Dataset Recognition

The detailed comparison results of single dataset recognition are presented in Table 6. The comparison results show that DSTNet achieves the highest F1-score in the classification tasks across all three single datasets, specifically reaching 0.7245 on SMIC, 0.7424 on CASME II, and 0.7186 on SAMM. These achievements highlight the outstanding performance in recognizing various ME categories, which is particularly crucial when dealing with class-imbalanced datasets. Furthermore, DSTNet also attains Acc of 0.7134 and 0.7426 on the SMIC and SAMM datasets, respectively, further demonstrating its superiority and reliability. Although on the CASME II dataset, DSTNet’s Acc is slightly lower than that of GEME and AUGCN + AUFusion, this is mainly because DSTNet focuses more on improving the overall recognition rate of each category rather than solely pursuing the highest accuracy. Overall, DSTNet exhibits good performance across all datasets.

#### 4.4.2. Composite Dataset Recognition

The detailed comparison results for composite dataset recognition are shown in Table 7. On the CDE dataset, DSTNet outperforms others, achieving the highest UF1 of 0.7979, with 20.97% and 19.73% improvements in UF1 and UAR over LBP-TOP, respectively. On CASME II, DSTNet also achieves the top UF1 of 0.9287 and the second UAR of 0.9232. Compared with SelfME, although DSTNet is slightly lower in terms of UAR, it increases the UF1 by 2.09%. Moreover, DSTNet outperforms the optical flow-based Bi-WOOF method, enhancing UF1 and UAR by 14.82% and 12.06%, respectively. Compared to STSTNet, which also uses a multi-stream design, DSTNet improves UF1 and UAR by 9.05% and 5.46%. On the SAMM dataset, DSTNet’s UF1 is second only to AUGCN + AUFusion. Meanwhile, it is noted that adaptive DSTNet performs slightly poorer than DSTNet overall on the CDE, CASME II, and SAMM datasets. This could be attributed to the low top-1 confidence of ME recognition models. However, on the lower-quality SMIC dataset, adaptive DSTNet shows the best performance, outperforming all other methods. This suggests that the adaptive strategy enhances model robustness with complex data, particularly benefiting subsequent cross-dataset recognition tasks.

#### 4.4.3. Cross-Dataset Recognition

The cross-dataset recognition results are detailed in Table 8. Adaptive DSTNet outperforms all other methods on the SMIC and CASME II datasets in all metrics, showcasing its strong generalization and robustness. On SAMM, while adaptive DSTNet achieves a marginally lower Acc compared to RN and micro-attention methods, it demonstrates substantially superior performance in terms of UAR, with improvements of 7.7% and 9%, respectively. This performance discrepancy suggests that while RN and micro-attention may achieve higher Acc due to overfitting to specific expression categories, they fail to maintain balanced recognition across all classes. In contrast, adaptive DSTNet has a more balanced performance in recognizing different categories of MEs, effectively reducing the missed and false detections. Compared to basic DSTNet, adaptive DSTNet consistently improves all evaluation metrics, validating the adaptive strategy’s effectiveness in complex data environments, especially for cross-dataset recognition.

### 4.5. Model Comparison with Other Methods

In the field of ME recognition, previous researches often focus on improving recognition performance while overlooking the significance of model size, such as the number of parameters and computational complexity. This brings notable drawbacks: large models not only increase computational resource consumption but also limit their applicability in resource-constrained environments. To comprehensively evaluate a model’s overall performance, it is essential to consider both its recognition performance and computational efficiency. Given that some studies do not provide the original code, we replicated the codes based on the network architectures described in the literature or tested the provided trained model to measure the model’s metrics, including the number of parameters, FLOPs, and results of the CASME II dataset, with a detailed comparison presented in Table 9.

The comparison results demonstrate that our proposed DSTNet and adaptive DSTNet achieved a reasonable balance between model size and recognition performance. Compared to methods with numerous parameters and complex computations, such as DSSN and ELRCN, DSTNet/adaptive DSTNet attains superior recognition results with only 3.15 M parameters and 64.77 M FLOPs. On CASME II, DSTNet/adaptive DSTNet exhibits stable recognition performance for both single-dataset Acc and composite-dataset UF1 metrics, even surpassing other lightweight methods like micro-attention and RCN. Although STSTNet has a smaller model size than our proposed method, its performance is significantly lower. On CASME II in the composite dataset recognition, STSTNet achieves the Acc of only 0.8382, whereas our proposed method achieves 0.9287. Furthermore, its performance in cross-dataset tests attests to its excellent generalization capability. In summary, our proposed method not only provides an efficient and lightweight solution for the field of ME recognition but also showcases the potential to achieve high-accuracy performance in practical applications.

## 5. Conclusions

In this study, we addressed the various issues in ME recognition by proposing targeted and feasible solutions. We designed a lightweight network named DSTNet, which is aimed at enhancing the performance of ME recognition. The motion magnification module serves as a virtual sensor enhancer to overcome physical sensor limitations, while the MDOF transforms raw sensor data into standardized spatio-temporal representations for efficient analysis. It is validated across various tasks, including single dataset, composite dataset, and cross-dataset scenarios. DSTNet exhibits efficient feature extraction capabilities, and the adaptive strategy devised in accordance with the network’s traits genuinely and effectively enhances the performance of ME recognition in complex tasks.

Addressing the challenges posed by the subtlety of movement and scarcity of data in ME recognition, we constructed a motion magnification network based on transfer learning. This network magnifies the facial muscle movements in apex frame images and introduces a magnification factor, which not only significantly enhances the facial muscle movement features closely associated with ME but also effectively expands the dataset. This method successfully overcomes the difficulties in extracting weak muscle movements from original images, achieving more precise spatial feature extraction. For brevity of duration in MEs, we applied a dense optical flow method to the onset frame and the magnified apex frame to capture the motion changes in ME sequences, i.e., MDOF. MDOF highlights the motion differences between frames, and by feeding it into the temporal stream for feature extraction, it delicately depict the subtle changes in muscle movement across frames. Combining the motion states and motion evolution features of ME facial muscle, we designed DSTNet, a dual-stream network architecture capable of simultaneously learning spatial and temporal representations to capture more discriminative information. Results on both single datasets and composite datasets demonstrate the effectiveness of DSTNet, particularly as evidenced by its F1-score, which underscores DSTNet’s exceptional performance in handling imbalanced data tasks.

To bolster the robustness of DSTNet, we also devised an adaptive strategy. For the most challenging cross-dataset recognition task in the field of ME recognition, we designed an adaptive strategy based on the top-1 confidence output from the model. The introduction of an adaptive magnification factor adjustment algorithm significantly enhances the robustness and generalization ability of the model in cross-dataset recognition tasks. Adaptive DSTNet achieves cross-dataset recognition results that surpass the state-of-the-art methods on both SMIC and CASME II. Furthermore, this simple yet effective strategy does not increase the size of recognition model and can serve as an inspiration for similar tasks.

However, it is important to acknowledge the limitations of this study. Despite the encouraging results, we obtained the facial muscle movement features of MEs in the quickest and simplest manner, and the model design is relatively lightweight, leaving room for improvement in model performance. In practical applications, the trade-off between model parameters and performance is crucial. Future work will focus on exploring alternative backbone architectures to improve model capability without compromising computational efficiency, as well as developing more sophisticated motion magnification techniques and adaptive magnification strategies to better capture subtle ME characteristics. Additionally, we aim to investigate the incorporation of multi-modal information, such as thermal imaging or non-contact physiological signals, to provide richer supplementary cues for ME recognition. Ultimately, our goal is to develop a comprehensive ME recognition system that can effectively function in practical applications.

## Figures and Tables

**Table 1:** The architecture of DSTNet.

Spatial Network (SN)			Temporal Network (TN)		
**Input: Magnified Apex Frame**			**Input: MDOF**		
**Layers**	**Parameters**	**Output Dimension**	**Layers**	**Parameters**	**Output Dimension**
Conv 1	size = 3×3, stride = 2	128×128×64	Conv 1	size = 5×5, stride = 2	126×126×16
Pooling 1	size = 2×2, stride = 2	64×64×64	Pooling 1	size = 2×2, stride = 2	63×63×16
Residual block 1	block = 2 size = 3×3, stride = 2	32×32×64	Conv 2	size = 3×3, stride = 1	61×61×16
Residual block 2	block = 2 size = 3×3, stride = 2	16×16×128	Pooling 2	size = 2×2, stride = 2	30×30×16
Residual block 3	block = 2 size = 3×3, stride = 2	8×8×256	Conv 3	size = 3×3, stride = 1	28×28×16
Pooling 2	size = 3×3, stride = 2	4×4×256	Pooling 3	size = 2×2, stride = 2	14×14×16
FC Layer	4096→500	500	FC Layer	3136→500	500
Feature fusion					
Output top-1 confidence					

**Table 2:** Results of model input ablation.

Methods	Acc	F1-Score
original apex + SN	0.5366	0.6011
magnified apex + SN	0.6057	0.5413
original dense optical flow + TN	0.5772	0.5427
MDOF + TN	0.6179	0.5874

**Table 3:** Results of data balance ablation.

Methods	Data Balance	Acc	F1-Score
magnified apex + SN	-	0.6057	0.5413
magnified apex + SN	✓	0.6220	0.6047
MDOF + TN	-	0.6179	0.5874
MDOF + TN	✓	0.6789	0.6793

**Table 4:** Results of SN and TN backbone depth ablation.

Methods	Blocks/Layers	Acc	F1-Score
magnified apex + SN + data balance	2	0.5935	0.5761
	3	0.6220	0.6047
	4	0.6057	0.5950
MDOF + TN + data balance	3	0.6789	0.6793
	4	0.6667	0.6634
	5	0.6341	0.6364

**Table 5:** Results of network structure ablation.

Structure	Input		Network Stream		Acc	F1-Score
	**Magnified Apex**	**MDOF**	**SN**	**TN**		
single-stream	✓	-	✓	-	0.6220	0.6047
	-	✓	-	✓	0.6789	0.6793
dual-stream *	✓	-	✓	✓	0.5854	0.5733
	-	✓	✓	✓	0.6911	0.6862
	✓	✓	-	✓	0.6829	0.6764
	✓	✓	✓	-	0.6341	0.6250
	✓	✓	✓	✓	0.7398	0.7424

* The last row indicates that the SN takes magnified apex as input, while the TN takes MDOF as input, which is DSTNet.

**Table 6:** Results of single dataset recognition. The best results are highlighted in bold, and the second-best results are underlined.

Methods	SMIC		CASME II		SAMM	
	Acc	F1-Score	Acc	F1-Score	Acc	F1-Score
FDM [36]	0.5488	0.5380	0.4593	0.4053	- *	-
Bi-WOOF + Phase [37]	0.6829	0.6700	0.6255	0.6500	-	-
Sparse MDMO [38]	0.7051	0.7041	0.6695	0.6911	-	-
SSSN [39]	0.6341	0.6329	0.7119	0.7151	0.5662	0.4513
DSSN [39]	0.6341	0.6462	0.7078	0.7297	0.5735	0.4644
Micro-attention [40]	0.4940	0.4960	0.6590	0.5390	0.4850	0.4020
GEME [41]	0.6463	0.6158	0.7520	$\underline{0.7354}$	0.5588	0.4538
AUGCN + AUFusion [24]	-	-	$\underline{0.7427}$	0.7047	$\underline{0.7426}$	$\underline{0.7045}$
KPCANet [42]	0.7174	$\underline{0.7100}$	0.7046	0.6952	0.6383	0.5215
**DSTNet (ours)**	$\underline{0.7134}$	0.7245	0.7398	0.7424	0.7426	0.7186

* signifies that this indicator is not reported in this literature.

**Table 7:** Results of composite dataset recognition. The best results are highlighted in bold, and the second-best results are underlined.

Methods	CDE		SMIC		CASME II		SAMM	
	UF1	UAR	UF1	UAR	UF1	UAR	UF1	UAR
LBP-TOP [35]	0.5882	0.5785	0.2000	0.5280	0.7026	0.7429	0.3954	0.4102
Bi-WOOF [35]	0.6296	0.6227	0.5727	0.5829	0.7805	0.8026	0.5211	0.5139
OFF-ApexNet [43]	0.7196	0.7096	0.6817	0.6695	0.8764	0.8681	0.5409	0.5392
CapsuleNet [44]	0.6520	0.6506	0.5820	0.5877	0.7068	0.7018	0.6209	0.5989
Dual-Inception [45]	0.7322	0.7278	0.6645	0.6726	0.8621	0.8560	0.5868	0.5663
STSTNet [46]	0.7353	0.7605	0.6801	0.7013	0.8382	0.8686	0.6588	0.6810
RCN [47]	0.7052	0.7164	0.5980	0.5991	0.8087	0.8563	0.6771	0.6976
GEME [41]	0.7395	0.7500	0.6288	0.6570	0.8401	0.8508	0.6868	0.6541
AUGCN + AUFusion [24]	$\underline{0.7914}$	0.7933	$\underline{0.7192}$	$\underline{0.7215}$	0.8798	0.8710	0.7751	0.7890
FeatRef [48]	0.7838	$\underline{0.7832}$	0.7011	0.7083	0.8915	0.8873	0.7372	0.7155
SMBANet [49]	0.7440	0.7459	0.6914	0.6934	0.8970	0.8920	0.6021	0.6068
SelfME [50]	-	-	0.6972	0.7012	$\underline{0.9078}$	0.9290	-	-
**DSTNet (ours)**	0.7979	0.7758	0.7041	0.6953	0.9287	$\underline{0.9232}$	$\underline{0.7604}$	0.7054
**adaptive DSTNet (ours)**	0.7832	0.7815	0.7328	0.7289	0.8854	0.8762	0.7563	$\underline{0.7547}$

**Table 8:** Results of cross-dataset recognition. The best results are highlighted in bold, and the second-best results are underlined.

Methods	SMIC			CASME II			SAMM		
	Acc	UF1	UAR	Acc	UF1	UAR	Acc	UF1	UAR
LBP-TOP [51]	- *	-	-	0.232	-	0.316	0.338	-	0.327
3DHOG [51]	-	-	-	0.373	-	0.187	0.353	-	0.269
D3DCNN [52]	-	-	-	0.447	-	-	0.369	-	-
RN [53]	-	-	-	0.578	-	0.337	$\underline{0.544}$	-	0.440
Micro-attention [40]	-	-	-	0.584	-	0.341	0.559	-	0.427
TFMVN [54]	-	-	-	0.455	-	0.367	-	-	-
RSTR [55]	0.451	-	-	0.562	-	-	-	-	-
ATNET [56]	-	0.503	$\underline{0.524}$	-	0.631	0.643	-	0.450	0.458
LGAttNet [57]	-	-	-	0.670	0.661	0.645	-	-	-
TKRM [58]	0.524	0.525	-	0.662	0.594	-	-	-	-
Meta-CDMERF [59]	-	-	-	0.638	0.600	0.626	0.498	0.425	0.513
**DSTNet (ours)**	0.463	0.457	0.461	0.634	0.628	0.630	0.526	$\underline{0.491}$	$\underline{0.486}$
**adaptive DSTNet (ours)**	0.537	0.542	0.545	0.690	0.688	0.675	0.534	0.529	0.517

* signifies that this indicator is not reported in this literature.

**Table 9:** Results of model comparison with other methods.

Methods	Image Input Size	Params (M)	FLOPs (M)	Results on CASME II
DSSN [39]	224×224×3, 224×224×3	58.31	1131.20	0.7078 (Acc, single dataset)
Micro-attention [40]	224×224×3	5.90	- *	0.6590 (Acc, single dataset)
OFF-ApexNet [43]	28×28×2	2.77	-	0.8764 (UF1, composite dataset)
STSTNet [46]	28×28×3	0.00167	0.448	0.8382 (UF1, composite dataset)
RCN [47]	224×224×3	4.11	2041.05	0.8087 (UF1, composite dataset)
ELRCN [60]	224×224×3, 224×224×5	219	12,145.91	0.384 (Acc, cross-dataset)
Micro-attention [40]	224×224×3	4.74	867.46	0.584 (Acc, cross-dataset)
ATNET [56]	224×224×3	8.16	1086.61	0.631 (UF1, cross-dataset)
DSTNet/adaptive DSTNet (ours)	256×256×3, 256×256×3	3.15	64.77	0.7398 (Acc, single dataset) 0.9287 (UF1, composite dataset) 0.690 (Acc, cross-dataset) 0.688 (UF1, cross-dataset)

* signifies that this indicator is not reported in this literature.

## Data Availability

Data derived from public domain resources.

## References

[1] Li J., Dong Z., Lu S., Wang S.J., Yan W.J., Ma Y., Liu Y., Huang C., Fu X. (2022). CAS(ME)3: A third generation facial spontaneous micro-expression database with depth information and high ecological validity. IEEE Trans. Pattern Anal. Mach. Intell..

[2] Wang Z., Zhang K., Luo W., Sankaranarayana R. (2024). HTNet for micro-expression recognition. Neurocomputing.

[3] Wahid Z., Bari A.H., Gavrilova M. (2023). Human Micro-Expressions in Multimodal Social Behavioral Biometrics. Sensors.

[4] Zhou X., Jin K., Shang Y., Guo G. (2018). Visually interpretable representation learning for depression recognition from facial images. IEEE Trans. Affect. Comput..

[5] Porter S., Ten Brinke L. (2008). Reading between the lies: Identifying concealed and falsified emotions in universal facial expressions. Psychol. Sci..

[6] Weinberger S. (2010). Airport security: Intent to deceive?. Nat. News.

[7] Zhao G., Li X., Li Y., Pietikäinen M. (2023). Facial Micro-expressions: An overview. Proc. IEEE.

[8] Ranjan R., Patel V.M., Chellappa R. (2017). Hyperface: A deep multi-task learning framework for face detection, landmark localization, pose estimation, and gender recognition. IEEE Trans. Pattern Anal. Mach. Intell..

[9] Zhang K., Zhang Z., Li Z., Qiao Y. (2016). Joint face detection and alignment using multitask cascaded convolutional networks. IEEE Signal Process. Lett..

[10] Oh T.H., Jaroensri R., Kim C., Elgharib M., Durand F., Freeman W.T., Matusik W. Learning-based video motion magnification. Proceedings of the European Conference on Computer Vision (ECCV).

[11] Niu W., Zhang K., Li D., Luo W. (2022). Four-player GroupGAN for weak expression recognition via latent expression magnification. Knowl.-Based Syst..

[12] Choi Y., Choi M., Kim M., Ha J.W., Kim S., Choo J. StarGAN: Unified Generative Adversarial Networks for Multi-Domain Image-to-Image Translation. Proceedings of the IEEE Conference on Computer Vision and Pattern Recognition (CVPR).

[13] Zhou J., Sun S., Xia H., Liu X., Wang H., Chen T. (2023). ULME-GAN: A generative adversarial network for micro-expression sequence generation. Appl. Intell..

[14] Yu J., Zhang C., Song Y., Cai W. ICE-GAN: Identity-Aware and Capsule-Enhanced GAN with Graph-Based Reasoning for Micro-Expression Recognition and Synthesis. Proceedings of the 2021 International Joint Conference on Neural Networks (IJCNN).

[15] Liong S.T., Gan Y.S., Zheng D., Li S.M., Xu H.X., Zhang H.Z., Lyu R.K., Liu K.H. (2020). Evaluation of the spatio-temporal features and gan for micro-expression recognition system. J. Signal Process. Syst..

[16] Li Y., Huang X., Zhao G. (2021). Micro-expression action unit detection with spatial and channel attention. Neurocomputing.

[17] Gan Y.S., Lien S.E., Chiang Y.C., Liong S.T. (2024). LAENet for micro-expression recognition. Vis. Comput..

[18] Liu Y.J., Zhang J.K., Yan W.J., Wang S.J., Zhao G., Fu X. (2015). A main directional mean optical flow feature for spontaneous micro-expression recognition. IEEE Trans. Affect. Comput..

[19] Xia Z., Hong X., Gao X., Feng X., Zhao G. (2020). Spatiotemporal Recurrent Convolutional Networks for Recognizing Spontaneous Micro-Expressions. IEEE Trans. Multimed..

[20] Xie H.X., Lo L., Shuai H.H., Cheng W.H. AU-Assisted Graph Attention Convolutional Network for Micro-Expression Recognition. Proceedings of the 28th ACM International Conference on Multimedia.

[21] Zachary Teed J.D. RAFT: Recurrent All-Pairs Field Transforms for Optical Flow. Proceedings of the European Conference on Computer Vision (ICCV) Workshops.

[22] Wang Z., Yang M., Jiao Q., Xu L., Han B., Li Y., Tan X. (2024). Two-level spatio-temporal feature fused two-stream network for micro-expression recognition. Sensors.

[23] Zhang K., Huang Y., Du Y., Wang L. (2017). Facial expression recognition based on deep evolutional spatial-temporal networks. IEEE Trans. Image Process..

[24] Lei L., Chen T., Li S., Li J. Micro-Expression Recognition Based on Facial Graph Representation Learning and Facial Action Unit Fusion. Proceedings of the IEEE/CVF Conference on Computer Vision and Pattern Recognition.

[25] Pumarola A., Agudo A., Martinez A.M., Sanfeliu A., Moreno-Noguer F. GANimation: Anatomically-aware Facial Animation from a Single Image. Proceedings of the European Conference on Computer Vision (ECCV).

[26] Zhang Y., Xu X., Zhao Y., Wen Y., Tang Z., Liu M. (2023). Facial prior guided micro-expression generation. IEEE Trans. Image Process..

[27] Iman M., Arabnia H.R., Rasheed K. (2023). A review of deep transfer learning and recent advancements. Technologies.

[28] Lucey P., Cohn J.F., Kanade T., Saragih J., Ambadar Z., Matthews I. (2010). The extended cohn-kanade dataset (ck+): A complete dataset for action unit and emotion-specified expression. Proceedings of the 2010 IEEE Computer Society Conference on Computer Vision and Pattern Recognition-Workshops.

[29] Zhao G., Huang X., Taini M., Li S.Z., PietikäInen M. (2011). Facial expression recognition from near-infrared videos. Image Vis. Comput..

[30] Lyons M., Akamatsu S., Kamachi M., Gyoba J. (1998). Coding facial expressions with gabor wavelets. Proceedings of the Third IEEE International Conference on Automatic Face and Gesture Recognition.

[31] Aifanti N., Papachristou C., Delopoulos A. (2010). The MUG facial expression database. Proceedings of the 11th International Workshop on Image Analysis for Multimedia Interactive Services WIAMIS 10.

[32] Li X., Pfister T., Huang X., Zhao G., Pietikäinen M. (2013). A spontaneous micro-expression database: Inducement, collection and baseline. Proceedings of the 2013 10th IEEE International Conference and Workshops on Automatic Face and Gesture Recognition (FG).

[33] Yan W.J., Li X., Wang S.J., Zhao G., Liu Y.J., Chen Y.H., Fu X. (2014). CASME II: An improved spontaneous micro-expression database and the baseline evaluation. PLoS ONE.

[34] Davison A.K., Lansley C., Costen N., Tan K., Yap M.H. (2016). SAMM: A spontaneous micro-facial movement dataset. IEEE Trans. Affect. Comput..

[35] (2019). Micro-Expression Grand Challenge 2019 (MEGC 2019)-Recognition Challenge. https://facial-micro-expressiongc.github.io/MEGC2019/.

[36] Xu F., Zhang J., Wang J.Z. (2017). Microexpression identification and categorization using a facial dynamics map. IEEE Trans. Affect. Comput..

[37] Liong S.T., Wong K. (2017). Micro-expression recognition using apex frame with phase information. Proceedings of the 2017 Asia-Pacific Signal and Information Processing Association Annual Summit and Conference (APSIPA ASC).

[38] Yong-Jin L., Bing-Jun L., Yu-Kun L. (2018). Sparse MDMO: Learning a Discriminative Feature for Spontaneous Micro-Expression Recognition. IEEE Trans. Affect. Comput..

[39] Khor H.Q., See J., Liong S.T., Phan R.C., Lin W. (2019). Dual-stream shallow networks for facial micro-expression recognition. Proceedings of the 2019 IEEE International Conference on Image Processing (ICIP).

[40] Wang C., Peng M., Bi T., Chen T. (2020). Micro-attention for micro-expression recognition. Neurocomputing.

[41] Nie X., Takalkar M.A., Duan M., Zhang H., Xu M. (2021). GEME: Dual-stream multi-task GEnder-based micro-expression recognition. Neurocomputing.

[42] Feng W., Xu M., Chen Y., Wang X., Guo J., Dai L., Wang N., Zuo X., Li X. Nonlinear deep subspace network for micro-expression recognition. Proceedings of the 3rd Workshop on Facial Micro-Expression: Advanced Techniques for Multi-Modal Facial Expression Analysis.

[43] Gan Y.S., Liong S.T., Yau W.C., Huang Y.C., Tan L.K. (2019). OFF-ApexNet on micro-expression recognition system. Signal Process. Image Commun..

[44] Van Quang N., Chun J., Tokuyama T. (2019). CapsuleNet for micro-expression recognition. Proceedings of the 2019 14th IEEE International Conference on Automatic Face & Gesture Recognition (FG 2019).

[45] Zhou L., Mao Q., Xue L. (2019). Dual-inception network for cross-database micro-expression recognition. Proceedings of the 2019 14th IEEE International Conference on Automatic Face & Gesture Recognition (FG 2019).

[46] Liong S.T., Gan Y.S., See J., Khor H.Q., Huang Y.C. (2019). Shallow triple stream three-dimensional cnn (ststnet) for micro-expression recognition. Proceedings of the 2019 14th IEEE International Conference on Automatic Face & Gesture Recognition (FG 2019).

[47] Xia Z., Peng W., Khor H.Q., Feng X., Zhao G. (2020). Revealing the invisible with model and data shrinking for composite-database micro-expression recognition. IEEE Trans. Image Process..

[48] Zhou L., Mao Q., Huang X., Zhang F., Zhang Z. (2022). Feature refinement: An expression-specific feature learning and fusion method for micro-expression recognition. Pattern Recognit..

[49] Wang G., Huang S., Tao Z. (2023). Shallow multi-branch attention convolutional neural network for micro-expression recognition. Multimed. Syst..

[50] Fan X., Chen X., Jiang M., Shahid A.R., Yan H. SelfME: Self-supervised motion learning for micro-expression recognition. Proceedings of the the IEEE/CVF Conference on Computer Vision and Pattern Recognition.

[51] Yap M.H., See J., Hong X., Wang S.J. (2018). Facial micro-expressions grand challenge 2018 summary. Proceedings of the 2018 13th IEEE International Conference on Automatic Face & Gesture Recognition (FG 2018).

[52] Talluri K.K., Fiedler M.A., Al-Hamadi A. (2022). Deep 3D convolutional neural network for facial Micro-expression analysis from video images. Appl. Sci..

[53] Peng M., Wu Z., Zhang Z., Chen T. (2018). From macro to micro expression recognition: Deep learning on small datasets using transfer learning. Proceedings of the 2018 13th IEEE International Conference on Automatic Face & Gesture Recognition (FG 2018).

[54] Zhang M., Huan Z., Shang L. (2020). Micro-expression recognition using micro-variation boosted heat areas. Proceedings of the Chinese Conference on Pattern Recognition and Computer Vision (PRCV).

[55] Zong Y., Zheng W., Hong X., Tang C., Cui Z., Zhao G. Cross-database micro-expression recognition: A benchmark. Proceedings of the 2019 on International Conference on Multimedia Retrieval.

[56] Peng M., Wang C., Bi T., Shi Y., Zhou X., Chen T. (2019). A novel apex-time network for cross-dataset micro-expression recognition. Proceedings of the 2019 8th International Conference on Affective Computing and Intelligent Interaction (ACII).

[57] Takalkar M.A., Thuseethan S., Rajasegarar S., Chaczko Z., Xu M., Yearwood J. (2021). LGAttNet: Automatic micro-expression detection using dual-stream local and global attentions. Knowl.-Based Syst..

[58] Chen Z., Lu C., Zhou F., Zong Y. (2023). TKRM: Learning a Transfer Kernel Regression Model for Cross-Database Micro-Expression Recognition. Mathematics.

[59] Wang H., Zhou J., Liu X., Jia Y., Chen T. (2025). A cross-database micro-expression recognition framework based on meta-learning. Appl. Intell..

[60] Khor H.Q., See J., Phan R.C.W., Lin W. (2018). Enriched long-term recurrent convolutional network for facial micro-expression recognition. Proceedings of the 2018 13th IEEE International Conference on Automatic Face & Gesture Recognition (FG 2018).

